# Effects of Gelatinization of Enteral Nutrients on Human Gastric Emptying

**DOI:** 10.4021/gr2010.06.213w

**Published:** 2010-05-20

**Authors:** Naruo Kawasaki, Mitsuyoshi Urashima, Hironori Odaira, Takuji Noro, Yutaka Suzuki

**Affiliations:** a Department of Surgery, International University of Health and Welfare Hospital, Japan; b Department of Surgery, Jikei University School of Medicine, Japan; c Division of Clinical Research and Development, Jikei University School of Medicine, Japan

**Keywords:** Gelatinization, Gastric emptying, Absorption, ^13^C breath test, Enteral nutrition

## Abstract

**Background:**

Gastrointestinal side effects, particularly diarrhea, are still the main reasons for discontinuation of enteral nutrition. Gelatinization of liquid meal for the prevention of diarrhea has been reported as effective. The purpose of this study was to investigate the effects of gelatinization of liquid meal on gastric emptying.

**Methods:**

Ten healthy volunteers were studied two times, with 2-week interval between tests. The total calorific value was set at 225 kcal, and 3 test meals were prepared: liquid meal and 2 types of gelatinized meals. These 2 types of gelatinized meals are different viscosity. ^13^C-sodium acetate (100 mg) was thoroughly mixed, and exhaled air was sampled. The results of gastric emptying were expressed as the time of peak excretion (T_max_), and absorption was expressed as the area under the ^13^CO_2_ curve up to T_max_ (AUC-T_max_). At the same time, blood samples were collected to measure levels of blood glucose, insulin and gastrin.

**Results:**

The mean value of T_max_ were 52.0, 77.3 and 85.6 min. Compared to liquid meal, gastric emptying for gelatinized meals was significantly delayed. The mean value of AUC-T_max_ were 22.7, 28.7 and 33.7%dose, respectively, and no significant differences in absorption were seen. No significant differences existed in blood glucose, gastrin and insulin.

**Conclusions:**

Gelatinization of liquid meal delays gastric emptying. Gelatinized liquid meal may be useful for the management of diarrhea accompanied with enteral nutrition without influencing gastrointestinal hormone and blood glucose.

## Introduction

The patients, who cannot orally consume the necessary amount of nutrients, are expanding. In supplementary nutrition, nutrients are administered from a nasogastric tube or gastrostomy, but clinically relevant complications such as gastroesophageal reflux, aspiration pneumonia and diarrhea are likely to occur [1, 2]. Gastrointestinal side effects, particularly diarrhea, are still the main reasons for discontinuation of enteral nutrition. The composition and osmotic pressure of enteral nutrition solution is frequently suspected of playing a leading role in causing diarrhea [3]. The management of diarrhea requires knowledge of gastro-intestinal physiology during enteral nutrition.

While gelatinization of liquid meal for the prevention of these complications has been reported as effective [4], no systematic assessments or scientific evidence support the efficacy of this approach. We used the ^13^C breath test to ascertain the effects of liquid meal and gelatinized meals on gastric emptying, absorption and gastrointestinal hormones.

## Materials and Methods

### Subjects and test meals

Subjects comprised 10 healthy volunteers [3 men, 7 women; age 28.6 ± 6.7 years; BMI, 20.6 ± 1.7 (mean ± SD)] who underwent the ^13^C breath test after 12 hours of fasting with ≥ 1 week of break between each test. Enteral nutrients were prepared using liquid meal (semi-digested nutritional supplement, 1 kcal/ml; Racol, Otsuka pharmaceutical, Tokyo, Japan) and Easygel gelatinizing agent, consisting of pectin and calcium lactate (total calorific value: 25 kcal; Otsuka pharmaceutical, Tokyo, Japan). Informed consent was obtained from all subjects prior to enrollment in this study.

The two nutritional formulas were well balanced for nitrogen and electrolyte. The total calorific value of each test meal was set at 225 kcal. Table 1 shows the composition of each test meal: (a) Liquid meal: 225 ml; (b) Gelatinized meal 1:200 ml of liquid meal and 25 kcal of gelatinizing agent (viscosity: 16,000 ± 800 cp); (c) Gelatinized meal 2:175 ml of liquid meal and 50 kcal of gelatinizing agent (viscosity: 128,000 ± 4,300 cp).

**Table 1:** Composition of Test Meals

Ingredient	Liquid meal /225 kcal	Gelatinized meal I /225 kcal	Gelatinized meal II /225 kcal
Protein (g)	9.855	9.06	8.265
Fat (g)	5.0175	4.46	3.9025
Glucide (g)	35.145	36.04	36.935
V.A (IU)	465.75	414	362.25
V.D (IU)	30.6	27.2	23.8
V.B1 (mg)	0.855	0.76	0.665
V.B2 (mg)	0.55125	0.49	0.42875
V.B6 (mg)	0.84375	0.75	0.65625
niocin (mg)	5.625	5	4.375
Pantothenic acid (mg)	2.1555	1.916	1.6765
Falia acid (µg)	84.375	75	65.625
V.B12 (µg)	0.72	0.64	0.56
V.C (mg)	63.225	56.2	49.175
V.K (µg)	140.625	125	109.375
V.E (mg)	1.4625	1.3	1.1375
Biotin (µg)	8.685	7.72	6.755
Na (mg)	166.05	379.6	593.15
Cl (mg)	263.25	234	204.75
K (mg)	310.5	276	241.5
Mg (mg)	43.425	38.6	33.775
Ca (mg)	99	134	169
P (mg)	99	159	219
Fe (mg)	1.40625	1.25	1.09375
Mn (µg)	299.25	266	232.75
Cu (µg)	281.25	250	218.75
Zu (mg)	1.44	1.28	1.12
Se (µg)	5.625	5	4.375
Dietary fiber (g)	0	2.6	5.2
Water (g)	191.25	215.7	240.15

Enteral nutrients were prepared using liquid meal (1 kcal/ml; Racol, Otsuka pharmaceutical, Tokyo, Japan) and Easygel gelatinizing agent, consisting of pectin and calcium lactate (total calorific value: 25 kcal; Otsuka pharmaceutical, Tokyo, Japan). The total calorific value of each test meal was set at 225 kcal. Liquid meal 225 ml, gelatinized meal 1:200 ml of liquid meal and 25 kcal of gelatinizing agent (viscosity: 16,000 ± 800 cp) and gelatinized meal 2:175 ml of liquid meal and 50 kcal of gelatinizing agent (viscosity: 128,000 ± 4300 cp).

With each test meal, 100 mg of ^13^C-sodium acetate (^13^C-labeled compound) was thoroughly mixed. Viscosity of the test meal was measured using a Type B rotating viscometer (VDA; Shibaura System, Tokyo, Japan) after stirring for 2 min at 12 rpm. Measurements were taken in triplicate at 20 ± 2 °C.

Test meals were consumed within 5 min of preparation, and 20 ml of water was consumed to flush down any residual meal in the esophagus.

### Measurement methods

Expired air was sampled before and 5, 10, 15, 20, 30, 40, 50, 60, 75, 90, 105, 120, 135, 150, 165, 180, 210 and 240 min after intake. The labeled meal was emptied from the stomach, absorbed through the duodenum, metabolized in the liver and then exhaled. The amount of ^13^CO_2_ in expired air was measured using an UBiT-IR300 (Fukuda Denshi, Tokyo, Japan).

### Assessment indicators

#### Gastric emptying

According to the standard gastric emptying procedure in the ^13^C breath test as published by the Japan Society of Smooth Muscle Research, T_max_ (Time of the highest %dose on the ^13^CO_2_ curve) was used to assess gastric emptying.

#### Absorption

Area under the ^13^CO_2_ curve up to T_max_ (AUC-T_max_: %dose) was used to assess absorption.

#### Blood biochemistry

Blood glucose, insulin and gastrin were measured by collecting a blood sample before and 30, 60, 90, 120, 150 and 180 min after intake.

### Statistical analysis

The results were expressed as mean ± standard deviation (SD). The effects of intervention compared with control (liquid meal) were computed by repeated measure analysis of variance using STATA 8.0. Statistical significance was set at P < 0.05 (Stat View 5.0; SAS Institute Inc, USA).

## Results

### Gastric emptying

**Figure 1:**
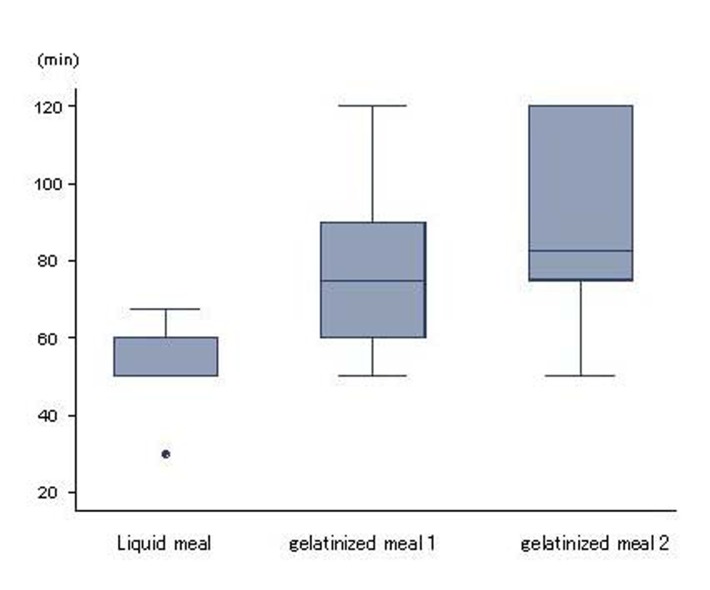
Gastric emptying. Liquid and gelatinized meals displayed differences in gastric emptying (p < 0.05). Higher viscosity was associated with slower gastric emptying. Results are expressed as mean ± SD.

Mean durations of gastric emptying for liquid meal, gelatinized meal 1 and 2 were 52.0 ± 10.1, 77.3 ± 20.8 and 85.6 ± 25.0 min, respectively. Compared to liquid meal, gastric emptying for gelatinized meals was significantly delayed (P < 0.05; Fig. 1).

### Absorption

**Figure 2:**
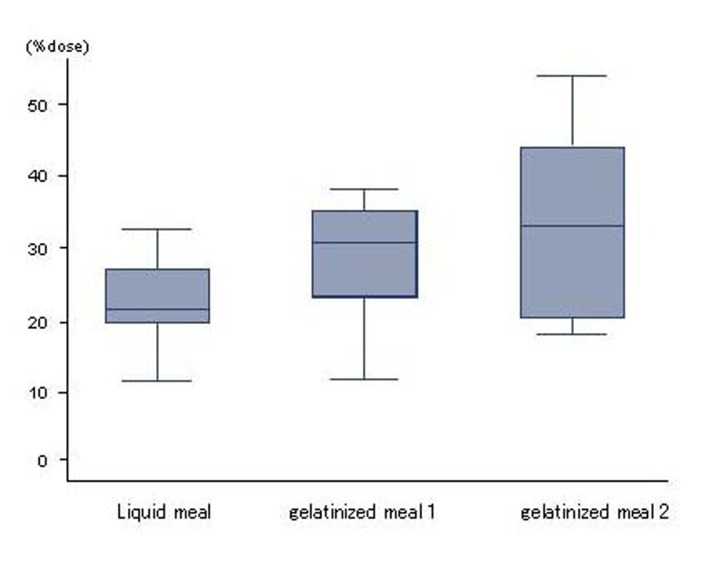
Absorption. No differences in absorption were seen between liquid and gelatinized meals. Results are expressed as mean ± SD.

The %dose for liquid meal, gelatinized meal 1 and 2 were 22.7 ± 6.1%, 28.7 ± 8.7% and 33.7 ± 12.7%, respectively, and no significant differences in absorption were seen between liquid and gelatinized meals (Fig. 2).

### Blood biochemistry

**Figure 3:**
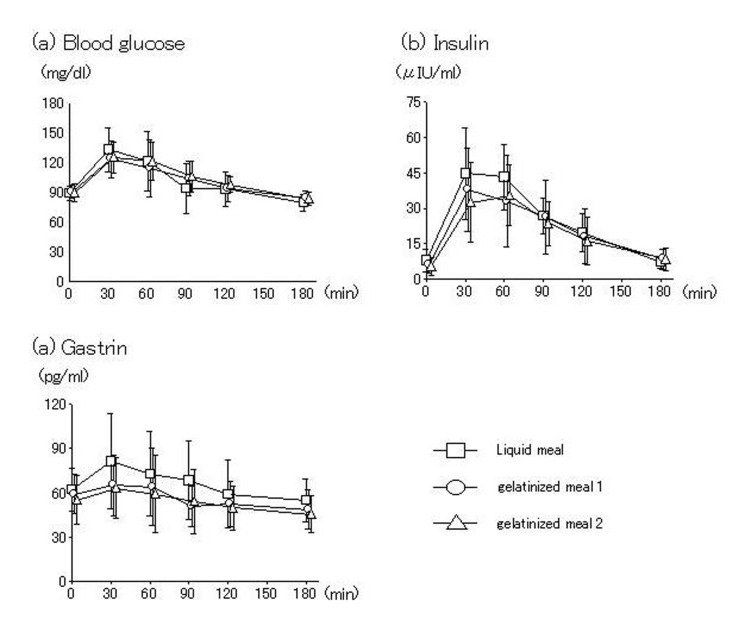
Blood glucose and gastrointestinal hormone. No differences in blood levels of glucose, insulin or gastrin were seen between liquid and gelatinized meals.

With all test meals, blood glucose and gastrin peaked 30 min after intake and insulin peaked 60 min after intake, then decreased over time. No significant differences among test meals were seen (Fig. 3).

## Discussion

The gelatinization of enteral nutrients is believed to be useful for preventing complications such as gastroesophageal reflux, aspiration pneumonia and diarrhea [1]. However, most past studies were empirical, and scientific evidence to support the efficacy of this approach has been lacking.

Gastric emptying can be measured directly by imaging modalities such as the radioisotope method and the opaque marker method, or indirectly by mixing a test meal and a reagent, such as acetaminophen, sulfamethizole or ^13^C, and allowing the mixture to be absorbed through the small intestine.

The ^13^C method is a gastric emptying test using a compound labeled with ^13^C, a non-radioactive stable isotope. Ever since the first report by Ghoos et al [5], this method has been performed widely, although mostly in Western countries [6, 7]. Also, the ^13^C method correlates closely with the radioisotope method, which is considered the golden standard for gastric emptying measurement, and is convenient and involves no radiation exposure [8, 9]. In Japan, studies have been conducted based on the standard guidelines published by academic societies such as the Japan Society of Smooth Muscle Research [10]. As a test meal, a liquid meal (200 kcal, 1 kcal/ml) thoroughly mixed with 10 mm of ^13^C-sodium acetate (^13^C-labeled compound) is recommended, and the present study followed this guideline.

When assessing gastric emptying, the duration for half of a test meal to empty (T1/2) [5] and the duration for ^13^CO_2_ in expired air to peak (T_max_) [6] have been used. Along a ^13^CO_2_ curve, duration to reach T_max_ is mostly related to gastric emptying and absorption of a ^13^C-labeled compound, while the time after T_max_ represents metabolism and excretion. In many cases, T1/2 is after T_max_, and when compared to T1/2, T_max_ is thought to more closely reflect gastric emptying due to the effects of the metabolism and excretion of a ^13^C-labeled compound.

In digestion and absorption tests using ^13^C-labeled compounds, long-chain fatty acids such as triolein, palmitic acid and trioctanoin, and their ester substrates, have been utilized [11-13], but digestion and absorption cannot be simultaneously assessed with gastric emptying. Most nutrients are absorbed through the small intestine, and in the stomach, nutrients are digested and then emptied. Gastric emptying and absorption should thus affect each other, and simultaneous assessment enables evaluation of gastrointestinal function. In the past, the concentration curve of a labeled-compound was used to calculate area under the entire curve and peak values of the labeled compound. However, the area under the entire curve also reflects the metabolism and excretion of a ^13^C-labeled compound. In addition, following upper gastrointestinal tract resection such as distal gastrectomy and increased gastric emptying can increase apparent absorption. Establishing indicators that are less likely to be affected by gastric emptying is thus necessary. We mentioned above that, from ^13^CO_2_ curves, the duration to reach T_max_ can be seen as the amount of time required to empty and absorb a ^13^C-labeled compound. Based on this notion, we assess food absorption based on the area under the curve up to T_max_.

Based on the present results, the gelatinization of liquid meal delays gastric emptying, but does not affect absorption or gastrointestinal hormone levels.

Gastric emptying is also markedly affected by calories [14], but is less affected by the composition and osmotic pressure of food [15]. In terms of food characteristics, liquid meals are emptied faster than solid meals [16], because of decreased relaxation of the proximal stomach [17, 18]. Gelatinization delays gastric emptying by transiently increasing the particle size of liquid meal, thus making breakdown of food more difficult. Gastric emptying of gelatinized liquid meal resembles that of solid meal.

Increased gastric emptying is thought to be related to diarrhea [19], and favorable absorption requires nutrients to come in contact with the small intestinal mucosa. While gelatinization does not affect absorption, gradual gastric emptying makes nutrients pass through the small intestine slower, thus preventing diarrhea.
